# Genome-Wide Mapping of Consanguineous Families Confirms Previously Implicated Gene Loci and Suggests New Loci in Specific Language Impairment (SLI)

**DOI:** 10.3390/children11091063

**Published:** 2024-08-30

**Authors:** Adnan Yousaf, Huma Hafeez, Muhammad Asim Raza Basra, Mabel L. Rice, Muhammad Hashim Raza, Muhammad Imran Shabbir

**Affiliations:** 1 Department of Biological Sciences, International Islamic University, Islamabad 45500, Pakistan; adnan.phdbt73@iiu.edu.pk (A.Y.); imran.shabbir@iiu.edu.pk (M.I.S.); 2 Speech-Language-Hearing: Sciences & Disorders, University of Kansas, Lawrence, KS 66045-7555, USA; mabel@ku.edu; 3 Centre for Clinical and Nutritional Chemistry, School of Chemistry, University of the Punjab, New Campus Lahore, Lahore 54590, Pakistan; humahafeez11@yahoo.com (H.H.); asimbasra.chem@pu.edu.pk (M.A.R.B.)

**Keywords:** genome-wide, parametric linkage analysis, specific language impairment, homozygosity mapping, loci

## Abstract

Specific language impairment (SLI) is a developmental disorder with substantial genetic contributions. A genome-wide linkage analysis and homozygosity mapping were performed in five consanguineous families from Pakistan. The highest LOD scores of 2.49 at 12p11.22-q11.21 in family PKSLI-31 and 1.92 at 6p in family PKSLI-20 were observed. Homozygosity mapping showed a loss of heterozygosity on 1q25.3-q32.2 and 2q36.3-q37.3 in PKSLI-20. A loss of heterozygosity mapped, in PKSLI-31 and PKSLI-34 flanks, *NFXL1* and *CNTNAP2*, which are genes previously identified in SLI. Our findings report novel SLI loci and corroborate previously reported SLI loci, indicating the utility of a family-based approach.

## 1. Introduction

Specific language impairment (SLI), which is also called developmental language disorder (DLD), is a prevalent communication disorder that hinders the development of language skills in typically developing children who have otherwise no developmental disabilities like hearing loss, autism disorder, and intellectual impairment. From now on, we will use the term SLI in this report. Among developmental disorders, SLI ranks as one of the most common, affecting around 7 to 10 percent of children in kindergarten. Notably, the impact of SLI typically endures throughout adulthood, emphasizing the long-term consequences of this disorder (NIDCD., 2019) [1]. Individuals with SLI may have trouble stringing words together to form sentences, learning new vocabulary, or using words correctly in conversation [2,3,4]. Children diagnosed with SLI face a heightened risk of experiencing lower academic achievement compared to their age peers. Additionally, they often encounter challenges in establishing social relationships. These difficulties can persist throughout their educational journey, with a higher likelihood of not progressing beyond high school completion [2,3,4]. Measuring expressive and receptive language skills can help understand language development among individuals [5]. A speech-language pathologist (SLP) can test language ability in children [6]. The SLP directly observes the child’s communication abilities in various contexts to gain insights into their speech and language skills [7]. Parents and teachers are involved in the evaluation process by providing valuable information through interviews and questionnaires [8]. The SLP assesses the child’s learning abilities to comprehensively understand their overall cognitive and academic skills, which can contribute to language development [9]. The SLP utilizes standardized tests to evaluate the child’s current language performance. These tests provide a standardized measure for comparing the child’s language skills with those of same-age peers [10].

A twin study estimated the concordance rate to be over 0.90 among monozygotic twins with SLI, indicating a genetic influence on this disorder [11]. A higher prevalence of SLI cases has been observed in the proband families compared to the control families, indicating the genetic inheritance of this impairment [8]. Identifying genetic factors involved in SLI is essential to understand the molecular basis of SLI. Genetic studies are extremely valuable in understanding neuronal connections, genetic counseling, and targeted early interventions in SLI. The pedigree analysis has shown a complex inheritance pattern [12]. This has made SLI challenging to sort out genes using classical techniques. Advances in human genetics have allowed us to study complex disorders [13]. Consanguineous families are essential in understanding genetic disorders in other complex genetic disorders [14]. Studying consanguineous families and modern advances in human genetics will be helpful [15] in unlocking genetic wonders linked with the SLI language. Several chromosomal loci have been identified, and candidate genes for SLI have been proposed [16,17,18,19,20,21]. However, these studies added a fraction of genetic understanding. Additional genetic studies using extended families are needed to interpret the previous findings and explore further gene loci involved in SLI.

Our study targets explicitly identified genetic loci associated with specific language impairment (SLI) in consanguineous families. Although studies on families with SLI-affected individuals suggest growing evidence of variable inheritance patterns, the underlying causes of this disorder remain unknown. Family studies are critical in identifying the molecular basis of SLI [20,22]. Linkage analysis has been used to study complex Mendelian disorders under different inheritance patterns, and this approach has also resulted in the successful mapping of the genetic loci of a complex genetic disorder, stuttering [23,24]. The pedigree-based analysis produced significant results in mapping genetic variants even with the undefined mode of inheritance and an incomplete disease penetrance model [25]. Different gene mapping methods have been used to reveal the genetics of SLI. Parametric and non-parametric linkage analyses and homozygosity mapping have been used to map genes in family studies of speech- and language-related disorders [16,19,22,24,26]. Parametric analysis is a model-based method to map genomic regions that co-segregate in the family. This method specifies the mode of inheritance, disease allele frequency, and disease penetrance. The analysis can be performed with an autosomal dominant or autosomal recessive inheritance model with complete or incomplete disease penetrance [22,27]. Non-parametric linkage is a model-free analysis, and it is used when several genes might contribute to the disease and when excessive sharing of the alleles is identical by descent in the linked loci [19]. Homozygosity mapping is a strong method to detect runs of consecutive homozygous genotypes in affected individuals in consanguineous families. In genetic disorders of the recessive mode of inheritance, which is common in consanguineous families, runs of homozygosity often carry a genetic variant shared among the affected individuals [28].

Pedigree-based analysis combining whole-genome linkage analysis and homozygosity mapping in 14 consanguineous Pakistani families revealed novel chromosomal loci in SLI [22]. The SLI Consortium (2002) used family-based linkage analysis for 98 families with language impairment and identified two susceptibility loci on chromosomes 16q24 and 19q13 [18,29]. A genome-wide statistically significant locus to 13q21 was observed in multiple families with language and reading impairment [16,30,31]. The association study in SLI identified a parent-of-origin effect. A significant paternal influence was observed on 14q12, and a suggestive maternal effect was identified on 5p13. This study provides a new dimension in understanding the genetics of SLI [32]. A family linkage study in Kansas identified a new linkage to 15q24.3-25.3 and confirmed 14q11.2-q13.3, indicating the utility of family studies in SLI [33].

Homozygosity mapping is widely used to identify candidate regions in recessive disorders. Homozygous regions arise from the inheritance of DNA segments from shared parents to their offspring [34]. In the study by Villanueva et al. (2011), homozygosity mapping was employed to analyze the Robinson Crusoe population, with the aim of identifying loci associated with specific language impairment (SLI) [19]. These Pakistani families provide opportunities to study the genetics of language impairment in Urdu-speaking consanguineous families, which are valuable to study.

The current study uses family history and the *Peabody Picture Vocabulary Test, Fourth Edition*, translated into Urdu (U-PPVT-4), to identify language impairment in five families. A parametric linkage analysis and homozygosity mapping were used to identify gene loci. This ongoing study confirms the gene loci reported in other families from the same population [22].

## 2. Materials and Methods

### 2.1. Selection of Individuals

The lack of available standardized assessments in Urdu led us to rely on family history information and receptive vocabulary performance in this sample. The children were initially identified from the Public Schools of Punjab, Pakistan. Information on how to identify children with language difficulties was provided to the schoolteachers. The study did not include children with obvious evidence of hearing loss, neurological diseases, or other developmental impairments. The idea is to identify children with the help of the school teacher based on their language abilities compared to their peers. This is a first step in identifying the probands for the subsequent family recruitment. After identifying probands, examiners from the University of Punjab visited the children and families by obtaining signed consent from the parents. First, proband parents completed the Rice Family History Questionnaire and Interview Form (PhenX Toolkit Protocol #200401) [32]. The form asked parents of the probands to respond to yes/no questions regarding the history of speech- and language-related difficulties and problems in learning to read, spelling, and general storytelling behaviors. A separate family history grid form was used to collect this information from all the available family members of the proband who consented to participate in this study.

The U-PPVT-4 was used to assess the receptive vocabulary of all the participating individuals aged 04 years one month to 53 years four months. All the items in U-PPVT-4 were translated into Urdu, a language spoken in Pakistan nationally. This translated version has been used to study Urdu-speaking individuals with typical and atypical language abilities [22,35]. For this test, the examiner pronounces a word, and the child selects a picture that best represents that word from a grid of four pictures [36]. The individuals were assigned with the affected or unaffected status based on their performance on the U-PPVT-4 using adjusted standard score cutoffs, which were previously applied to assign affection status in this sample for previous genetic analyses [22,37]. The adjusted U-PPVT-4 standard score cutoffs were less than 80 in males and less than 75 in females to account for expected literacy rate differences between sexes [22,38]. Additional information concerning the reliability and validity of this method in our clinical sample was provided in the previous publication [22].

**Figure 1:**
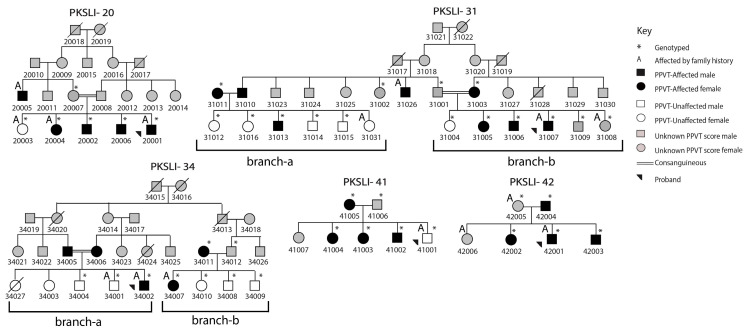
Families with specific language impairment (PKSLI). Large brackets underneath the younger generation of PKSLI-31 and PKSLI-34 are labeled with the branches for analysis.

The DNA was available from 41 individuals. However, 46 individuals from all the families were enrolled in this study. Based on the family information provided by parents, pedigrees were drawn for each family by representing males with squares and females with circles. The marriage is shown with a single line between parents and consanguineous marriages with a double line. The black-filled circles and squares indicate affected individuals with SLI, while unaffected individuals are shown with unfilled symbols, and the language data were not available for individuals with grey-filled symbols. The crossed line on the squares or circles shows the deceased individuals in the families. The U-PPVT-4 standard scores from 38 individuals were gathered, and based on this, 26 were affected, and 12 were unaffected (Figure 1).

The University of Kansas’s Institutional Review Board (IRB) approved this study with protocol # STUDY00143136. All individuals and parents of children signed the informed consent form to participate in the study; parents signed the consent form for children under 18. Oragene-Discover OGR-500 kits, Otawa, ON, Canada from DNA Genotek were used to collect the saliva samples from 41 individuals. The saliva samples were used to extract the DNA according to the manufacturer’s protocol (Genotek).

### 2.2. SNP Genotyping and Linkage Analysis

The Illumina Infinium QC Array-24, San Diego, CA, USA, which contains 15,949 SNPs, was used to perform SNP genotyping. The SNP genotyping was outsourced to Johns Hopkins University School of Medicine, Genetic Resources Core Facility. The subsequent genetic analysis was performed at the University of Kansas. The quality of the SNP data was checked in GenomeStudio 2.0 as per the guidelines provided by Illumina. The genotype data had an overall call rate of 99.86%. The genotype call quality was checked with the call rate of six CEPH samples used as positive controls during SNP genotyping. Out of 15,949 SNPs, 11,994 markers on autosomal chromosomes were used for linkage analysis and homozygosity mapping. The inheritance inconsistency (parent–child and parent–parent–child error) in two DNA samples was identified. These two DNA samples were excluded from the subsequent genetic analysis.

Single-point parametric linkage analysis was performed with SUPERLINK ONLINE SNP 1.1 [39]. Superlink-Online SNP 1.1 allows the performance of linkage analysis in large pedigrees while simultaneously analyzing thousands of SNP markers for single-point linkage analysis. The modes of inheritance were variable in all the families. Therefore, we performed linkage analysis on five families under autosomal dominant and recessive inheritance models and using a complete and incomplete disease penetrance. Using variable disease penetrance is recommended in calculating the Likelihood of Odds (LOD) scores during linkage studies of complex genetic disorders [24,40]. A disease allele frequency of 0.001 with a 0% phenocopy rate was used during the linkage analysis. In addition, a branch-wise linkage analysis was performed in two extended families (PKSLI-31, PKSLI-34). Both families were split into two branches, labeled as ‘a’ and ‘b’, assuming genetic heterogeneity within families [24]. The highest LOD scores are reported at the recombination fraction θ = 0. The human genome assembly GRCh37/hg19 was used to report the genomic locations of the SNP markers with the highest LOD scores.

### 2.3. Homozygosity Mapping

The HomozygosityMapper (http://www.homozygositymapper.org/), accessed on 1 May 2022, was used to perform homozygosity mapping, which provides the sizes of homozygous regions among affected individuals [28]. The analysis was performed independently for five families. The unaffected individuals in the PKSLI families were used as controls for this analysis. The branch-wise analysis was completed in PKSLI-31 and PKSLI-34. The marker allele frequency of zero and an option of genetic homogeneity were chosen in the HomozygosityMapper. The homozygous regions were excluded if more than 20 markers showed the homozygous regions in the controls [28]. The informative homozygous regions were selected against these parameters for further analysis. Each homozygous region was analyzed manually. The homozygosity regions were individually compared among affected and unaffected individuals. If the homozygosity was observed in multiple unaffected individuals, those regions were not considered for further analysis. All homozygosity regions identified in this study were cross-referenced with those previously reported for SLI in the Pakistani population [22].

## 3. Results

Two families, PKSLI-20 and PKSLI-31, showed positive LOD scores >1. However, we did not observe LOD scores >1 in three families, PKSLI-34, PKSLI-41, and PKSLI-42 (Table 1; Supplementary Table S2). Under the recessive mode of inheritance with complete disease penetrance, the highest LOD score of 1.92 was identified at chromosome 6p21.1-p12.3 in PKSLI-20. The highest scoring markers in this region spanning 5.3 Mb were rs736794 (chr6 = 40,997,833) and rs 1372567 (chr6 = 46,362,078). The highest LOD score of 2.49 on chromosome 12p11.22-p11.21 (2.9 Mb) was observed in PKSLI-31 under the dominant mode of inheritance with complete disease penetrance. The highest scoring marker in this analysis was rs581642, with a LOD score of 2.49 (Supplementary Table S2). The highest LOD score of 1.87 on 12q13.11-q13.12 was obtained in PKSLI-31 branch-a under the dominant mode of inheritance and complete disease penetrance.

**Figure 2:**
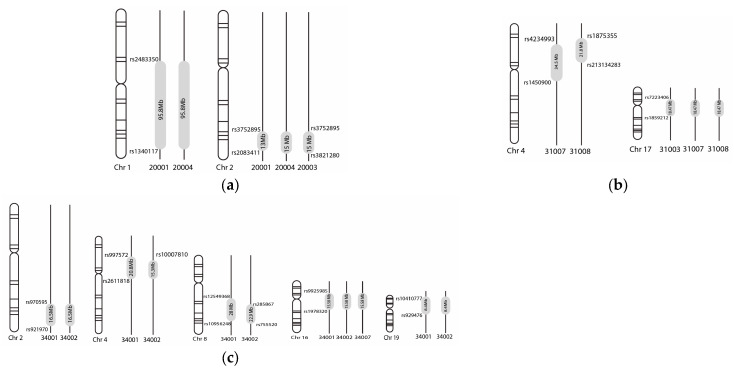
Loss of heterozygous loci in each family are labeled (**a**) PKSLI-20, (**b**) PKSLI-31, (**c**) PKLSI-34. The grey-shaded area in vertical lines shows each family member’s loss of heterozygosity regions. The family IDs and chromosome numbers are labeled under the vertical lines and chromosomes. The size of the loci is labeled in Megabases (Mb) in the grey-shaded area, and the SNP markers show the distal and proximal boundaries of the loci.

Multiple regions of loss of heterozygosity were observed in PKSLI-20, PKSLI-31, and PKSLI-34 (Figure 1). The SNP markers showing heterozygous genotypes define the boundaries of exciting homozygosity regions (Figure 2).

## 4. Discussion

The current study reports regions on chromosomes 2, 4, 7, 8, and 17 aligned with prior research, and our study proposed additional new loci on chromosomes 1, 6, 12, 16, and 19. Our findings emphasize the effectiveness of the family-based approach in identifying novel loci and confirming existing genetic factors associated with SLI.

Two-point and multi-point linkage analysis in PKSLI-20 revealed the highest LOD score of 1.92 at 6p21.1-p12.3 under the recessive mode of inheritance and complete penetrance (Supplementary Table S1). The linkage region 6p21.1-p12.3 spans 5.3 Mb in PKSLI-20, housing 656 genes. We observed *TM4SF20* in the loss of heterozygous locus, 2q36.3-q37.3 (rs3752895-rs3821280) in PKSLI-20, indicating an opportunity to reproduce these findings [41]. The *TM4SF20* is associated with early language delay and white matter hyperintensities (WMH) phenotypes reported in a study of 15 probands with language delay phenotype [41]. This locus was also reported in an unrelated Pakistani SLI family, indicating a promising region for future study [22].

Multiple linkage loci were observed in PKSLI-31, with the highest single-point LOD score of 2.49 to 12p. Multiple loci in one family support the hypothesis of polygenic inheritance in SLI [18]. Another linkage region (7q35-q36.1 in PKSLI-31), a loss of heterozygosity region (4p15-p14 in PKSLI-31 and PKSLI-34), overlaps with the previously reported regions in an isolated Chilean family, showing promise for the identification and confirmation of candidate genes [19]. The critical candidates in these regions are *CNTNAP2* and *NFXL1,* which are strongly implicated in SLI [19].

An incomplete co-segregation of the loss of heterozygosity regions was noted in all the families. One example is that individual 34001 was identified as affected with language impairment based on the U-PPVT-4 score, but they share the loss in the heterozygosity region with the affected sibling, 34002. The family report shows a history of language impairment in this individual, indicating the need to establish a standardized assessment of U-PPVT-4 in the Urdu-speaking population. We are in the process of collecting the U-PPVT-4 data from multiple age groups of the Urdu-speaking population, which may provide a population-based language assessment and scoring threshold for language impairment in Urdu-speaking individuals.

## 5. Conclusions

In conclusion, this study used linkage and homozygosity mapping analysis to evaluate the genomic data of consanguineous Pakistani families. Novel Loci linked to SLI phenotype were found. The homozygosity region may have the potential to find candidate genes related to SLI. Multiple loci in family PKSLI-20 and PKSLI-31 can be due to multigene involved for SLI phenotype. Focusing on linked loci and narrowing down homozygous regions can help find candidate genes. There could be a possible language assessment bias using the PPVT-4 in the non-English speaking population. Although we addressed this bias to some extent by adjusting the standard score based on gender and culture, there is a need for a study to define the age-based norms of U-PPVT-4 in the Urdu-speaking population. We have an ongoing data collection of the U-PPVT-4 from the Pakistani population in different age groups. In the future, these data can be useful in precisely defining the age-matched cut-points for language assessment. Future studies may include the confirmation of these loci and performing whole exome sequencing in these families to identify genes and associated variants at these reported loci.

## Figures and Tables

**Table 1:** The candidate linkage loci observed in PKSLI families according to full penetrance.

Family/Branch	Chromosome	Linkage Loci (hg 19) *	Snip ID	Maximum LOD Score	Mode of Inheritance
PKSLI-20	6	6p21.1-p12.3	rs736794	1.92	recessive
PKSLI-31	12	12p11.22-q11.21	rs581642	2.49	dominant
PKSLI-31-a	12	12q13.11-q13.12	rs581642	1.87	dominant
PKSLI-31	7	7q35-q36.1	rs6464094	2	recessive

* hg19: human genome assembly 19 (February 2009).

## Data Availability

The data presented in this study are available on request from the corresponding author to protect the privacy of the participants.

## Supplementary Materials

The following supporting information can be downloaded at: https://www.mdpi.com/article/10.3390/children11091063/s1, Table S1. Single-point LOD scores in PKSLI-20 at 6p21.1-p12.3 under the recessive mode of inheritance and complete penetrance. Table S2. Single-point LOD scores in PKSLI-31 on 12p11.22-p11.21 (2.9 Mb) under the dominant mode of inheritance and complete penetrance. Table S3. Linkage scores in PKSLI-31 on 12q13.11-q13.12 under the dominant mode of inheritance and complete penetrance. Table S4. Single-point LOD scores in PKSLI-31 on 7q35-q36.1 (5.2 Mb) under the recessive inheritance model and complete penetrance.
