# Supplementary material for: Genome-Wide Mapping of Consanguineous Families Confirms Previously Implicated Gene Loci and Suggests New Loci in Specific Language Impairment (SLI)
Source: Children (Basel). 2024 Aug 30;11(9):1063. doi: 10.3390/children11091063 (PMC11429814; doi:10.3390/children11091063)
Supplement: Supplementary file 1 [file children-11-01063-s001.zip › children-3029484-supplementary.pdf]

Supplementary Tables

Supplementary Table S1. Single-point LOD scores in PKSLI-20 at 6p21.1-p12.3 under the recessive mode of inheritance and complete penetrance

SNP ID	Genomic Location	LOD Score
rs13196885	36.408	-2.2743
rs4711531	38.026	1.2739
rs910516	38.34	1.0256
rs1738240	38.539	1.0256
rs1885615	39.113	1.024
rs9349128	39.563	1.024
rs736794	40.773	1.9259
rs1011101	41.418	1.024
rs722269	41.97	0.7259
rs1537638	42.871	1.2739
rs2270860	43.045	1.0256
rs1563788	43.083	1.0256
rs3778492	43.421	1.0256
rs945131	43.491	0.7259
rs1322669	44.254	1.024
rs227850	44.474	1.0256
rs1293457	44.533	1.0256
rs3778507	44.672	1.0256
rs1928533	45.285	1.024
rs9472516	45.337	0.7259
rs995564	45.858	1.2739
rs4714910	46.007	1.0256
rs1372567	46.104	1.9259
rs953887	46.137	1.9259
rs2277119	46.385	1.0256
rs9381468	46.4	1.0256
rs953062	46.401	1.0256
rs993612	47.019	1.0256
rs1556358	47.825	0.7259
rs1991355	48.383	0.7259
rs9369842	48.77	1.0256
rs2032553	48.908	1.0256
rs1471539	49.235	0.0983
rs9463517	49.35	1.024
rs1480617	49.356	1.2739
rs7760223	49.367	1.024
rs9463520	49.367	1.2739
rs1923523	50.454	1.024
rs283548	50.741	1.024
rs283545	50.743	1.024
rs1327264	50.944	1.0256
rs2397060	51.386	1.0256
rs966707	51.403	1.0256
rs875142	52.003	1.024
rs2180314	52.393	1.2739

rs4715332	52.444	0.0983
rs4715354	52.484	1.2739
rs405729	52.618	0.7259
rs7496	52.618	1.2739
rs367836	52.618	0.7259
rs9370288	53.962	-1.0745

Supplementary Table S2. Single-point LOD scores in PKSLI-31 on 12p11.22-p11.21 (2.9Mb) under the dominant mode of inheritance and complete penetrance

SNP ID	Genomic Location	LOD Score
rs7973582	27.517	-3.85
rs10506029	28.179	0.7799
rs4931123	28.85	1.4468
rs10843425	29.327	-2.2065
rs2043623	29.333	-2.2065
rs759890	29.535	-2.3653
rs1909160	29.851	0.4564
rs1909148	29.871	0.4564
rs1622965	30.133	0.8601
rs581642	30.717	2.4918
rs256722	30.729	-1.6367
rs11051174	30.798	1.1633
rs1666228	31.737	1.7003
rs325437	32.228	1.0053
rs904582	32.441	-1.8302
rs1473652	32.509	-1.8302
rs4931655	32.764	1.084
rs1353933	32.85	-2.2065
rs1705748	33.839	0.37
rs1705772	33.88	0.37
rs2068119	34.465	1.606
rs10880624	38.232	0.37
rs1906260	38.306	-0.0554
rs1843910	38.383	-1.5674
rs12298504	39.305	-0.0554
rs956066	40.002	1.902
rs1005955	40.046	-0.0554

Supplementary Table S3. Linkage scores in PKSLI-31 on 12q13.11-q13.12 under the dominant mode of inheritance and complete penetrance

SNP ID	Genomic Location	LOD Score
rs759890	29.535	-2.0953
rs1909160	29.851	0.6929
rs1909148	29.871	0.6929
rs1622965	30.133	0.994
rs581642	30.717	1.8798
rs256722	30.729	-2.1078
rs11051174	30.798	0.6929
rs1666228	31.737	1.0596
rs325437	32.228	0.994
rs904582	32.441	-2.2365
rs1473652	32.509	-2.2365
rs4931655	32.764	1.3937
rs1353933	32.85	-2.2119
rs1705748	33.839	0.1762
rs1705772	33.88	0.1762
rs2068119	34.465	0.994
rs10880624	38.232	0.1762
rs1906260	38.306	0.1762
rs1843910	38.383	-2.2365
rs7970273	38.752	-2.2365
rs12298504	39.305	0.1762
rs956066	40.002	1.8798
rs1005955	40.046	0.1762
rs1820545	40.516	0.994
rs2920816	40.569	0.994
rs872168	41.865	-2.2365
rs2061192	42.381	1.3937
rs12322164	44.119	0.994
rs1495042	45.14	-2.2365
rs2731032	45.183	-2.2365
rs965125	45.428	-2.1078
rs1012642	46.059	-2.2365
rs1444588	46.17	0.994
rs1492891	46.306	-2.2365
rs2471583	46.627	-2.2365
rs832723	47.319	0.994
rs10875671	47.723	-2.2365
rs7970314	48.014	0.6929
rs1978161	48.173	1.8798
rs1542707	48.341	1.8798
rs1107654	48.387	1.8798
rs1274726	49.207	0.994
rs296736	50.047	-1.9412

Supplementary Table S4. Single-point LOD scores in PKSLI-31 on 7q35-q36.1 (5.2 Mb) under the recessive inheritance model and complete penetrance

SNP ID	Genomic Location	LOD Score
rs700273	145.298	-0.6208
rs1524341	145.828	0.7023
rs73472817	146.477	1.3791
rs2710084	146.777	-0.0635
rs963314	146.984	-0.0353
rs243491	147.567	0.7023
rs6464094	149.536	2.0003
rs1547958	149.762	0.2027
rs875588	150.29	1.6999
rs2536077	150.53	-0.8985
rs10235893	151.3	0.1836
rs6951978	152.009	-0.0096
